# The Role of Somatic Mutation in Hereditary Hemorrhagic Telangiectasia Pathogenesis

**DOI:** 10.3390/jcm14134479

**Published:** 2025-06-24

**Authors:** Evon DeBose-Scarlett, Douglas A. Marchuk

**Affiliations:** Molecular Genetics and Microbiology, Duke University Medical Center, Durham, NC 27710, USA

**Keywords:** hereditary hemorrhagic telangiectasia, somatic mutation, second hit, mosaic

## Abstract

Historically, the factor(s) that stimulate vascular malformation genesis in hereditary hemorrhagic telangiectasia (HHT) has been hotly debated. Once heterozygous loss-of-function germline mutations in *ENG*, *ACVRL1*, or *SMAD4* were discovered in individuals with HHT, haploinsufficiency, a 50% reduction in the encoded protein, was proposed as the molecular mechanism of HHT. However, the focal and discrete nature of HHT-associated vascular malformations suggested to others that vascular malformation genesis requires an additional, local trigger. In this review, we discuss the evidence for the Knudsonian two-hit mutation mechanism of vascular malformation pathogenesis in HHT, where the inherited, heterozygous mutation is augmented by an acquired somatic mutation in the remaining normal copy of the gene. We consider the mechanisms of HHT–vascular malformation development in the broader context of the emerging role of somatic mutations in both sporadic and inherited vascular malformations. We discuss different mechanisms of biallelic gene inactivation in HHT, difficulties with the detection of all possible mechanisms of biallelic inactivation, and issues related to the somatic mosaic nature of the lesion. We then discuss the critical importance of non-genetic factors on the pathogenesis of HHT-associated vascular malformations. Finally, we discuss the implications of the two-hit mutation mechanism for the design of novel treatments for HHT.

## 1. Introduction

Hereditary hemorrhagic telangiectasia (HHT) is an inherited disorder characterized by vascular malformations [1]. Telangiectases typically occur on the skin, mucous membranes, and liver. Rupture of these telangiectases causes nosebleeds and gastrointestinal bleeding, which can lead to anemia. Larger arteriovenous malformations (AVMs) occur in the internal organs, typically the liver, lungs, and brain. AVMs, abnormal direct connections between arteries and veins bypassing the normal capillary bed, cause much of the morbidity of HHT. Hypoxia, high output heart failure, brain abscess, internal hemorrhage, stroke, and even death can result. The major treatment for HHT-associated AVMs remains embolization or surgery [2].

HHT is inherited in an autosomal dominant pattern. Individuals inherit a heterozygous loss-of-function mutation in *ENG*, *ACVRL1*, or *SMAD4* [3,4,5]. *ENG* or *ACVRL1* mutations are found in approximately eighty-five percent of patients, with approximately equal proportions of each gene [6]. *SMAD4*, which causes a combined syndrome of juvenile polyposis–HHT (JP-HHT), accounts for approximately two percent of positive genetic diagnoses [6]. More recently, an “HHT-like” phenotype has been described in individuals with mutations in *GDF2*, encoding BMP9, but it is unclear whether the phenotype of these patients should be clinically categorized as HHT [7,8,9,10,11]. Importantly, these proteins all function in the bone morphogenetic protein (BMP)9/10 signaling pathway in endothelial cells [6]. Activation of this pathway is thought to promote vascular quiescence [12,13,14]. BMP9/10 signaling is also implicated in several other endothelial cell functions, including organ-specific endothelial cell differentiation, endothelial cell polarity and migration, and mural cell recruitment [15].

## 2. The Search for the Etiology of HHT-Associated AVMs: Non-Genetic Mechanisms

Initial investigations into the etiology of HHT-associated vascular malformations focused on the expression of endoglin (encoded by *ENG*) and ALK1 (the commonly used term for the protein encoded by *ACVRL1*) in monocytes and neonatal human umbilical vein endothelial cells (HUVECs) from affected individuals [16,17,18,19,20]. These studies found reduced expression of endoglin or ALK1 on the surface of cells of affected individuals, and only transient, intracellular expression of the mutant protein. This suggested that haploinsufficiency was the mechanism of HHT pathogenesis. However, this hypothesis does not fully explain the phenotype of HHT, which is characterized by multiple, focal vascular malformations rather than a systemic vascular defect. This discrepancy between genotype and phenotype spurred the investigation into additional factors that may contribute to the etiology of HHT-associated vascular malformations.

Non-genetic factors had long been thought to drive AVM formation. Earlier theories of AVM development involved the persistence of developmental arteriovenous connections that normally disappear and failures in capillary development [21]. A hierarchical model of AVM development proposes that because arterial sprouts give rise to lower flow veins, mechanical defects in this process, in conjunction with single nucleotide polymorphisms in angiogenesis-related genes, lead to the formation of AVMs [22]. While this model proposed the involvement of genetic factors, these factors were considered secondary to mechanical processes.

## 3. Somatic Mutations in Sporadic Vascular Malformations: Gain-of-Function Mutations

Somatic mutations have long been known to contribute to disease initiation and progression. Their role in cancer has been firmly established [23,24], but they are also well-known to contribute to other types of diseases [25]. In the past two decades, somatic mutation has also been identified as a key factor in the formation of vascular malformations. Somatic gain-of-function mutations in *TEK* and *PIK3CA* were discovered in venous malformations [26,27]. A gain-of-function mutation in *MAP3K3* was identified in sporadic cerebral cavernous malformations (CCMs) [28]. Even AVMs, long thought to be primarily determined by improper maintenance of high blood flow, were found to harbor somatic mutations. Somatic gain-of-function mutations in *KRAS* were identified in brain AVMs [29]. These findings were supported by additional studies in brain AVMs and extracranial AVMs, which identified gain-of-function mutations in RAS/RAF/MEK/ERK pathway genes, including *MAP2K1*, *BRAF*, and *KRAS* in AVMs [30,31,32,33]. More recently, mosaic *KRAS* mutation was shown to be sufficient to cause vascular malformations in mice and zebrafish [34]. These studies demonstrated the importance of somatic mutation as a primary cause of AVMs.

Most studies elucidating the role of somatic mutations in vascular malformations focused on sporadic, non-inherited vascular malformations. Sporadic malformations are typically caused by a single gain-of-function mutation in a driver gene. Gain-of-function mutations are often confined to single base mutations at specific codons causing specific amino acid changes or precise indels affecting specific protein regulatory or enzymatic domains. The gain-of-function mutations found in vascular malformations are also often the identical oncogenic mutations also identified in tumors. These mutations are found specifically in the vascular malformation tissue and are not present in constitutional DNA samples from the patient, confirming their somatic nature. Due to their restriction to specific codons within the gene that would lead to enhanced activity, gain-of-function mutations are comparatively rarer in the genome than loss-of-function mutations. However, the recurrence of the same mutation in multiple vascular malformation samples of the same classification facilitates their identification and validation. Conversely, loss-of-function mutations may occur at almost any region within a gene, including exons, splice junctions, untranslated regions, and even introns. They can span from single base substitutions to whole gene deletions. Thus, there are many more ways to genetically inactivate a gene than to activate one.

Inherited vascular malformations are typically caused by germline loss-of-function mutations. Patients inherit a heterozygous loss-of-function mutation in a specific gene, with the mutation present in every cell, including every endothelial cell lining the blood vessels in their body. The difference in mutation type between sporadic and inherited malformations is also reflected in the presentation of these lesions in affected individuals. Individuals with sporadic vascular malformations typically have at most one or two lesions. Individuals with inherited malformations typically present with multiple vascular malformations, and their lesion burden increases with age. The identification of somatic mutations in sporadic vascular malformations raised the question of whether somatic mutations may also contribute to the development of inherited forms of these vascular malformations.

## 4. Somatic Mutations in Inherited Vascular Malformations: The Genetic Two-Hit Hypothesis

**Figure 1:**
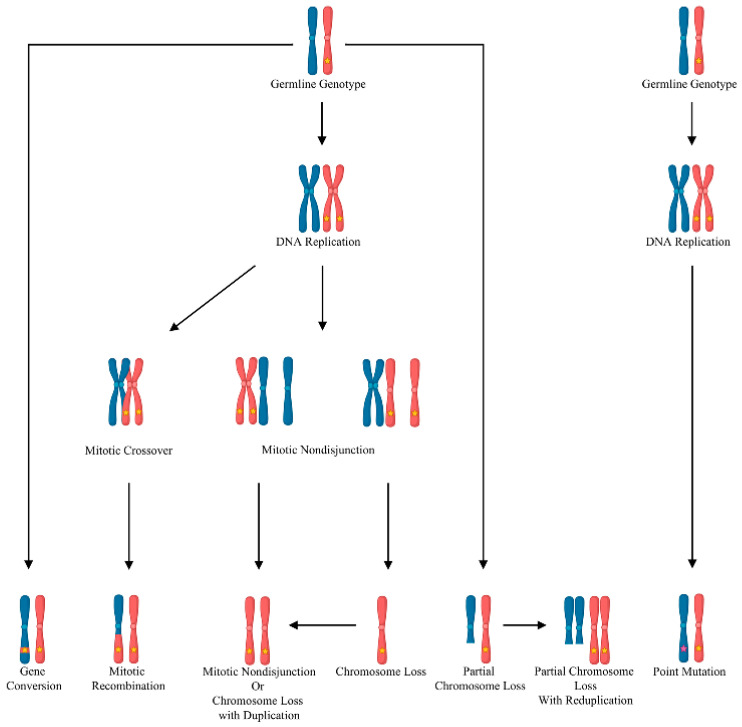
Mechanisms of somatic gene inactivation. Mechanisms of somatic loss of heterozygosity resulting in loss of wild-type allele (blue) and homozygosity or hemizygosity for germline mutation (yellow star). Genetic analysis would show only chromosome of germline mutation (red) over region of loss of heterozygosity, which may be relatively small (Gene Conversion) or extend from point of mitotic recombination to end of chromosome (Mitotic Recombination) or lead to loss of whole chromosome (Chromosome Loss with Duplication). Somatic point mutation of wild-type allele (purple star) may also result in biallelic gene inactivation. In this case, germline and somatic mutations are distinct, and somatically mutant cells retain their constitutional zygosity at all loci. Created in BioRender (2025).

In contrast to haploinsufficiency, in which a germline mutation causing the loss of one allele is sufficient to cause dysfunction, the Knudsonian two-hit mutation mechanism can explain the multi-focal and discrete nature of the vascular malformations in inherited disorders. The Knudsonian model hypothesizes that, in individuals harboring a heterozygous germline loss-of-function mutation, the remaining wild-type allele is inactivated by somatic mutation or loss of heterozygosity (Figure 1). This causes local biallelic loss of gene function in a small number of cells. These biallelically mutant cells seed the development of the vascular malformation [35]. This mechanism has been well-established in cancer. Evidence supporting the role for somatic loss-of-function mutations in inherited vascular malformations has been found in multiple disorders. Biallelic germline and somatic loss-of-function mutations in *KRIT1*, *CCM2*, or *PDCD10* were identified in inherited CCMs [36,37,38]. A somatic loss-of-function mutation in *TEK* causing local loss of the wild-type allele in combination with a weakly activating germline mutation in the same gene was discovered in a venous malformation from an individual with inherited mucocutaneous venous malformation [27]. Even in inherited AVMs, germline and somatic loss-of-function mutations in *RASA1* were identified in AVM tissue from an individual with capillary malformation–arteriovenous malformation 1 [39,40]. This growing body of research established somatic mutations as a critical component of both sporadic and inherited vascular malformation disorders.

## 5. Evidence for Two-Hit Mutation Mechanism in HHT: Clinical Observations

Clinical observations of the progression and location of telangiectases suggest a possible role for somatic mutation in their development. One study reported a patient with daily sun exposure to the left forehead with increased telangiectases in that area [41]. Ultraviolet (UV) exposure is a well-known cause of somatic mutation, suggesting that UV-induced somatic mutations over the course of a person’s lifetime may lead to mucocutaneous telangiectases on exposed surfaces of the body. The probability of external triggers causing somatic mutation is likely to differ between tissues owing to anatomical factors and exposure to mutagens. The skin is more highly exposed to potential mutagens, such as UV from sun exposure, than the brain. Similarly, the liver is also relatively exposed, as it clears the blood of waste and harmful chemicals in its normal function. These substances may be potential mutagens causing somatic mutation in an internal organ context. Trauma and toxins may stimulate the quiescent endothelium to proliferate, leading to acquired somatic mutations. However, indirect evidence alone is not confirmation of somatic mutations as a required mutational event in HHT.

## 6. Evidence for Two-Hit Mutation Mechanism in HHT: Preclinical Models

Preclinical models of HHT further provide evidence of the requirement of biallelic loss of an HHT gene to develop vascular malformations. Initial attempts to create mouse models of HHT demonstrated the importance of biallelic loss of HHT gene function in the pathogenesis of HHT-associated vascular malformations. Mice homozygous for null mutations in *Eng* and *Acvrl1* were created to elucidate the role of these genes in the HHT phenotype. Complete deletion of *Eng* and *Acvrl1* in mouse models causes embryonic lethality at E10–10.5 due to defects in angiogenesis [42,43,44,45,46]. The deletion of *Smad4* causes a failure of mesoderm formation [47,48]. These developmental phenotypes confirmed that all three genes have essential roles in cardiovascular development.

Thus, the first live mouse models of HHT were *Eng* and *Acvrl1* heterozygotes, reflecting the patient constitutional genotype. *Eng* and *Acvrl1* heterozygous mice showed only a mild and variable HHT phenotype after extended observation [42,49,50]. In these heterozygous models, the genetic two-hit model was never tested. The variable expressivity and age of clinical symptom onset reflects clinical characteristics of HHT patients. However, these mice generally showed low penetrance, and did not reliably reproduce the major HHT phenotype of interest, AVMs. This demonstrated that haploinsufficiency for an HHT gene was insufficient to generate AVMs.

Cre-lox technology was used to cause inducible deletion of both copies of *Eng* or *Acvrl1* in mouse models. This proved the key to creating a reproducible AVM phenotype in mice. Similarly, biallelic loss of *eng* or *acvrl1* in zebrafish is necessary to generate AVMs [51,52]. Even “vessel-on-a-chip” models of HHT using human endothelial cells require biallelic loss of *ACVRL1* to develop arteriovenous shunts in vessel organoids [53,54]. Across multiple models of HHT, including in vitro models and in vivo models of different species, complete loss (both copies) of an HHT gene is required to generate an AVM.

## 7. HHT-Associated Vascular Malformations: Testing the Two-Hit Mutation Hypothesis

When *ENG* was identified as the first HHT gene, the two-hit mutation mechanism was proposed as a potential molecular mechanism for vascular malformations associated with HHT [3]. But subsequent investigations into endoglin and ALK1 expression on cells from HHT patients argued against a local biallelic loss-of-function mechanism [16,17,18,19,20]. However, recent developments in deep, next generation sequencing technologies allowed for more thorough investigation of this hypothesis in human HHT-associated vascular malformation samples. In 2019, somatic mutations causing biallelic loss of *ENG* or *ACVRL1* function were identified in HHT-associated telangiectases [55]. Low variant allele frequency somatic mutations were identified in the same gene as the germline mutation in approximately half of mucocutaneous telangiectases studied. These somatic mutations were confirmed to occur on the wild-type allele, thus causing biallelic loss-of-function specifically in the telangiectasis tissue. Subsequently, somatic mutations causing loss-of-function in *ENG* or *ACVRL1* were identified in HHT-associated AVMs of internal organs [56,57]. In addition, loss of heterozygosity affecting whole chromosome or chromosome arms was identified in vascular malformations that lacked a somatic point mutation, indicating that somatic gene inactivation could be caused by multiple genetic mechanisms [57]. Later, somatic mutation and loss of heterozygosity causing biallelic loss-of-function in *SMAD4* was identified in an AVM from a JP-HHT patient, confirming that all three major HHT genes follow the same biallelic inactivation mechanism to cause AVMs in humans [58]. Recently, somatic mutations in *ACVRL1* causing biallelic loss-of-function were found in liver vascular malformations and a skin telangiectasis from one patient, providing further evidence of biallelic HHT gene inactivation in HHT-associated vascular malformations [59]. These recent findings in HHT join the wider body of literature, solidifying the critical role of somatic mutations underlying the development of hereditary AVMs in HHT.

## 8. What Cell Type Acquires Somatic Mutations?

Current sequencing efforts of human HHT-associated vascular malformations have focused on bulk affected tissue isolated from HHT patients. To date, no study has attempted to isolate individual cells from a vascular malformation to determine the cellular origin of the somatic mutation. However, mouse models for all three HHT genes provide evidence that endothelial cells acquire somatic mutation. Complete loss of *Eng* or *Acvrl1* was required specifically in endothelial cells to cause AVMs. Experiments using endothelial cell-specific Cre drivers could cause AVMs, while those targeting vascular smooth muscle cells, macrophages, or pericytes could not [60,61,62,63,64,65,66]. Thus, animal models suggest that the pathogenesis of the AVM begins with the endothelial cell.

## 9. How Much Protein Function Must Be Lost to Cause AVMs?

Although the exact level of loss of protein function to initiate AVM pathogenesis has yet to be determined, endoglin, ALK1, or SMAD4 protein expression must be reduced to some threshold below 50%. In HHT, the evidence in preclinical models shows that a 50% reduction in expression due to a germline heterozygous mutation is not sufficient to induce AVMs.

In an autosomal dominant disorder caused by an inherited mutation, the expression level of the relevant gene is constrained by the fact that humans carry two copies of each gene in each cell. Loss of gene expression due to mutation is expected to occur in a stepwise fashion in 50% increments due to loss of each gene copy. The simplest molecular mechanism for reducing gene expression below this threshold for a cell containing a heterozygous mutation in an HHT gene is loss of the remaining wild-type allele through a somatic mutation. This would be true for any autosomal dominant disease caused by an inherited loss-of-function mutation.

Nonetheless, certain germline mutations may have a larger effect on total protein function than others. Certain missense mutant endoglin proteins can form heterodimers with wild-type endoglin in the endoplasmic reticulum. This reduces trafficking of wild-type endoglin to the cell membrane, potentially exacerbating the effects of the heterozygous mutation on the loss of endoglin function in mutant cells [67]. Similarly, protein truncating variants (i.e., frameshift and nonsense mutations) in endoglin can exacerbate cellular stress and thereby reduce endothelial cell function [68]. These studies suggest that specific germline mutations may reduce protein function below 50%, even in the heterozygous state, and this may contribute to HHT phenotype severity. However, even in these patients, vascular malformations present as discrete lesions rather than systemic vascular dysplasia. This demonstrates that regardless of the category of inherited mutation, the threshold for AVM pathogenesis lies well below the 50% level of protein function. Inactivation of the remaining wild-type allele is the most likely molecular mechanism to explain the reduction in HHT–protein expression or function to initiate an AVM.

Evidence for non-genetic mechanisms to lower signaling capacity is found in both mouse models and clinical observations. Non-genetic mouse models of HHT using BMP9/10 blocking antibodies also show retinal AVMs, akin to those produced by endothelial-specific loss of HHT genes [13,69,70]. Strikingly, there have been several recent clinical reports of pulmonary arterial hypertension (PAH) patients administered the drug sotatercept exhibiting HHT-like signs, including epistaxis, telangiectases, and pulmonary right to left shunting [71,72,73,74,75]. Sotatercept, an activin ligand trap, reduces pro-proliferative transforming growth factor beta (TGFB) signaling, rebalancing TGFB/ BMP signaling toward the anti-proliferative BMP pathway [76]. However, measurements of circulating biomarkers revealed that sotatercept reduced circulating BMP9 and BMP10 levels in patients after twenty-four weeks [77]. If sotatercept also sequesters BMP9 and BMP10, this “off-target” effect could reduce endothelial BMP9/10 signaling and, much like the BMP9/10 blocking antibody mouse models of HHT, lead to the HHT-like phenotype seen in these PAH patients. Thus, these clinical observations highlight a possible example of non-genetic acquisition of HHT-like vascular malformations due to a reduction in BMP9/10 signaling. However, the fact remains that for HHT patients, already carrying an inherited mutation that reduces BMP9/10 signaling, the most likely molecular mechanism for further reduction in this signaling pathway is inactivation of the remaining wild-type copy of the HHT gene through somatic mutation.

## 10. Somatic Mutation Mosaicism

Protein staining for endoglin or Alk1 in brain AVMs from mouse models of HHT showed that AVMs caused by inducible loss of *Eng* or *Acvrl1* are mosaic for cells lacking expression of the protein [78,79]. Sequencing of human HHT-associated vascular malformations exhibits low variant allele frequencies of somatic mutations [55,56,57,58]. These data support the hypothesis that AVMs comprise a mix of somatically mutant (knockout) and non-somatically mutant (heterozygous) endothelial cells. A key question resulting from this observation is what proportion of endothelial cells in a pre-lesional vessel are required to be somatically mutated to initiate an AVM. Studies in wild-type mice transplanted with endothelial-specific *Acvrl1* knockout bone marrow-derived endothelial cells showed that increasing the proportion of *Acvrl1* negative cells by increased tamoxifen dosage led to larger brain AVMs [79]. A similar correlation between the number of *Eng* knockout endothelial cells and AVM severity was observed in an *Eng* mouse model [80]. A vessel-on-a-chip model was only able to reliably generate AVM-like shunts when *ACVRL1* knockout cells were seeded with wild-type cells in a 1:1 ratio, suggesting that the starting proportion of somatically mutant endothelial cells necessary to generate AVMs is relatively high [54]. However, in an *Eng* mouse model, only a minority of endothelial cells in brain AVMs lacked endoglin expression [61,78]. This evidence instead suggests that only a small proportion of the endothelial cells in an AVM are mutant. One potential explanation is that knockout cells clonally expand before dysplastic vessels remodel to incorporate non-mutant endothelial cells [79]. This temporal and mosaic mechanism of vascular malformation development is also seen in CCMs [81,82]. Thus, the pre-lesional vessel may contain a high proportion of HHT gene knockout cells due to the local expansion of a somatically mutant clone. However, non-somatically mutant endothelial cells are later incorporated into the vessel as it remodels, thereby reducing the proportion of knockout endothelial cells in the developing AVM.

Notably, all the aforementioned models combine knockout cells (biallelic loss-of-function) onto a wild-type background endothelium. This does not reflect the true state of an HHT patient where the underlying endothelium is heterozygous for the germline mutation. These heterozygous mutant endothelial cells undoubtedly contribute to the AVM. The true genetic model of the HHT-associated AVM would involve biallelic mutant cells present in a heterozygous background. Thus, the true percentage of somatically mutant endothelial cells necessary to initiate an AVM in an HHT patient may be quite different than these models suggest.

## 11. Barriers to Identifying Somatic Mutations

A major criticism of the necessity of somatic mutations for the development of HHT-associated vascular malformations has been that studies of somatic mutations in human vascular malformation tissue invariably fail to identify somatic genetic inactivation in every study sample [55,56,57]. This leaves the question of whether somatic mutation is a requirement of vascular malformation development, or if it is a contributing factor to some but not all vascular lesions unanswered.

However, not all mutations are readily identified by standard investigation methods. In fact, not all germline mutations, present at 50% allele frequency, are identified, even in clinically definitive cases of HHT. Loss of heterozygosity, large deletions, insertions, or rearrangements encompassing multiple exons or even whole genes are not easily identified using clinical mutation identification methods. In addition, noncoding mutations that alter gene expression are not captured in the typical gene panel sequencing that focuses on coding regions. Somatic mutation and loss of heterozygosity are even more difficult to identify due to the low variant allele frequency found in vascular malformation tissue. The variant allele frequency often falls below 5%, such that somatic mutation identification requires ultra-deep DNA sequencing and specialized computational algorithms.

Additionally, the ability to identify somatic mutations in vascular malformation tissue is heavily dependent on sample quality and purity. Endothelial cells are the only cell type expected to harbor the relevant somatic mutation, and only a minority of endothelial cells in the AVM are expected to be somatically mutant. The vascular endothelial cells line the inside of the malformed vessel in a single layer, like the latex surrounding a water balloon. They comprise a deceptively small proportion of what might be an already miniscule tissue sample. Vascular malformations from the liver and lung are often removed from the whole explanted organ, including much of the surrounding organ parenchyma. Mucocutaneous telangiectases removed from punch biopsies also include surrounding skin tissue. The loss of blood flow to the malformation makes even expert identification of the vascular lesion extremely difficult, and even the most careful excision of the vascular malformation still includes some non-endothelial cells from the surrounding tissue. The contribution of these non-endothelial cells to the DNA sample effectively lowers the apparent variant allele frequency of any somatic mutation, making it even more difficult to identify.

Even brain AVMs, which are surgically removed with as little accompanying brain tissue as possible, are not exempt from these sample acquisition considerations. A large portion of a surgically resected AVM is required for histopathological analysis. Of the remaining tissue, the anatomical context of the arteries and veins within the vascular malformation is lost, making it difficult to determine whether the relevant part of the lesion is used for DNA extraction. This is an important factor since evidence suggests that mutant endothelial cells may be more concentrated on the venous side of the lesion [83,84,85]. Thus, which part of the lesion is tested for mutations has a profound influence on the ability to identify somatic mutations in AVM tissue.

In addition, surgical samples are often formalin-fixed and paraffin-embedded, with no unfixed tissue available for analysis. The fixation process causes DNA damage, resulting in poor DNA quality and sequencing artifacts [86]. Low quality sequencing templates and an abundance of sequencing artifacts increases the difficulty of identifying true somatic mutations. Thus, given the multiple barriers to identifying somatic genetic inactivation of HHT genes, somatic inactivation is likely a requirement for all HHT-associated vascular malformations, but a combination of technical and sample considerations makes it improbable that a somatic mutation can be identified in every HHT-associated vascular malformation sample.

Given these challenges to identify second-hit somatic mutations in HHT-associated vascular malformation tissue, ultra-deep (greater than 1000×) DNA sequencing using a targeted gene panel is the most effective currently available approach. A targeted panel should include HHT genes, as well as other genes associated with AVMs and vascular malformations. In clinically ambiguous cases where the diagnosis of HHT is not definitive, inclusion of other vascular malformation genes aids in establishing whether or not the lesion is associated with HHT. Specific DNA polymerases used during polymerase chain reaction (PCR) to generate DNA sequencing libraries can introduce stereotypic artifacts, resulting in a high number of spurious identified variants [87]. To combat this issue, one strategy is to generate two or three libraries for each sample using different DNA polymerases and consider only the variants identified in more than one library [87]. This ensures that identified variants are present on the template genomic DNA and are not technical artifacts introduced during library generation. The variant analysis tool used greatly impacts the variants that are identified. One strategy is to use multiple variant analysis tools since each tool uses a different algorithm and may not identify certain variants identified by other tools [87,88,89]. Computationally identified variants should be manually screened in a genome viewer such as Integrative Genomics Viewer (IGV) [90] to ensure they appear on clean, non-duplicate reads in both the forward and reverse directions. Additional validation by an independent round of sequencing or, ideally, an orthogonal method, such as allele-specific PCR or droplet digital PCR, confirms the validity of putative variants.

## 12. Other Mechanisms of Biallelic Loss-of-Function—Epigenetic Modification

Somatic mutation is not the only mechanism through which the wild-type allele can be silenced. Epigenetic mechanisms of gene silencing could cause loss of gene function while remaining undetected by standard DNA sequencing methods. DNA methylation is one such mechanism. DNA methylation typically refers to 5-methylcytosine, which is primarily found at CG dinucleotides (CpGs) in the human genome [91]. Although a well-known mechanism of gene inactivation in cancer, somatic promoter methylation causing loss of gene function is not limited to cancer [91,92,93,94]. Biallelic inactivation of *FDFT1* through somatic mutation and promoter methylation has been shown to cause porokeratosis, a disorder causing both inherited and sporadic forms of skin lesions [95]. Although to date, loss of HHT gene function through somatic promoter methylation of the wild-type allele has not been shown, this mechanism may explain a fraction of vascular malformations lacking either somatic mutation or somatic loss of heterozygosity.

## 13. Are There Exceptions to the Biallelic Mutation Mechanism in HHT?

HHT is caused by inherited loss-of-function mutations, and as predicted by the two-hit mutation hypothesis, all second-hit somatic mutations identified in HHT tissues also cause loss-of-function of the same gene as the germline mutation [55,56,57,58]. One unusual situation where it might appear uncertain whether two genetic hits are required is the recurrence of liver vascular malformations after liver transplantation in HHT patients. The cells from the liver donor would not contain the HHT patient’s germline mutation, eliminating the first hit required in the genetic two-hit hypothesis. Fluorescence in situ hybridization staining for X and Y chromosomes in biopsied liver tissue in donor/recipient sex mismatched cases demonstrated an increased number of host endothelial cells lining vascular channels in the transplanted liver [96]. Host endothelial cells in the blood vessels of the transplanted liver would be heterozygous for the patient’s disease-causing mutation and at similar risk of second-hit somatic mutation as other host liver endothelial cells. Thus, this conundrum of the development of AVMs in a liver from a non-HHT donor does not necessarily invalidate the mechanism of biallelic HHT gene inactivation in AVMs from HHT patients.

## 14. Frequency of Biallelic Gene Inactivation

**Figure 2:**
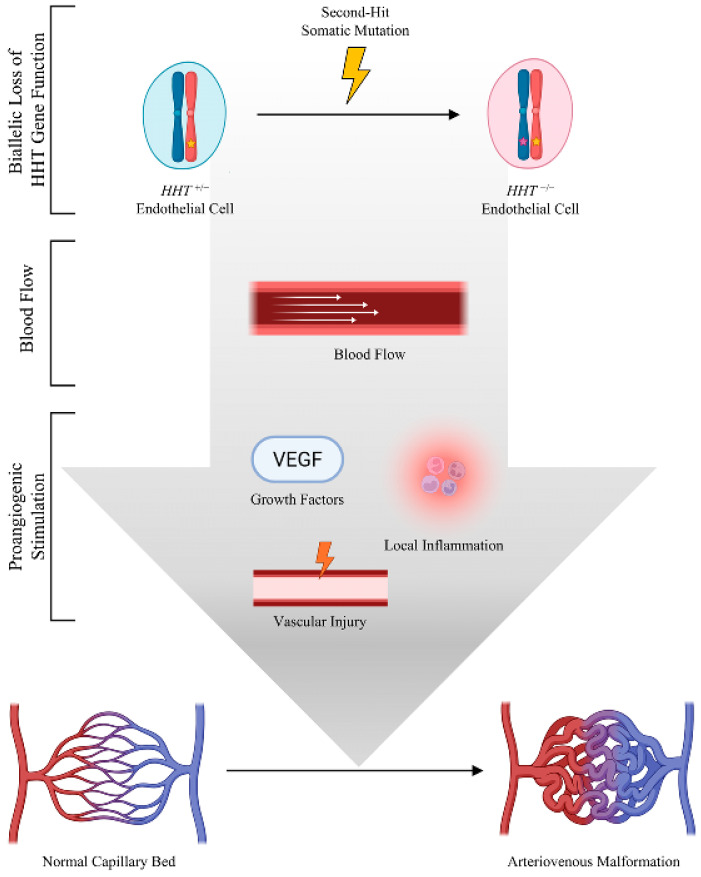
Multifactorial model of HHT-associated AVM development. Proposed model of HHT-associated vascular malformation pathogenesis in which biallelically mutant endothelial cells respond to hemodynamic cues and angiogenic stimulus inappropriately to develop arteriovenous malformation. Germline mutation (yellow star). Somatic inactivation of wild-type allele (purple star). Created in BioRender (2025).

Still, a major concern might be whether the frequency of somatic mutations is sufficient to account for the many vascular malformations observed in HHT patients. Using published estimates of the somatic mutation frequency per nucleotide per cell division, and the number of endothelial cells in an adult, Snellings et al. [55] calculated that an adult HHT patient harboring an *ENG* germline mutation would have 1.5 million endothelial cells with a somatic loss-of-function mutation in *ENG*. Significantly, this calculation only considered point mutations and small indels readily identified in standard next generation sequencing. Loss of heterozygosity over large chromosomal regions may also cause biallelic loss of gene function of HHT genes [57,58]. Estimates of loss of heterozygosity range from 10^−3^ to 10^−5^ per locus per cell division [97,98]. Combining the estimates of these two mutation mechanisms, an individual with HHT with a germline mutation in *ENG* would have millions of endothelial cells that harbor biallelic loss-of-function due to somatic mutation or loss of heterozygosity. Therefore, rather than a being a rare event, biallelic loss-of-function in the context of germline heterozygosity is extremely common. These estimates then present the opposite to the original concern about the frequency of somatic mutation. Instead of explaining how such a seemingly rare event occurring by chance could be necessary to cause AVMs, the frequency of biallelic inactivation compels the question of why, if somatic mutations cause AVMs, patients have maximally hundreds and not many millions of vascular malformations. A solution to this concern is that although somatic mutations are required, they alone are not sufficient to cause HHT-associated vascular malformations. Additional triggers including blood flow, angiogenic stimulation, and inflammation are necessary to stimulate AVM formation. However, only endothelial cells with biallelic loss-of-function respond to these triggers by forming an AVM (Figure 2).

## 15. Triggers of AVM Formation

The adult endothelium is considered a quiescent tissue with a low cell turnover rate under homeostatic conditions [99], suggesting that some external trigger is required to stimulate angiogenesis and cell proliferation in somatically mutant (knockout) endothelial cells.

Induced knockout of *Eng* or *Acvrl1* in addition to proangiogenic stimulus, such as vascular endothelial growth factor (VEGF) injection or lipopolysaccharide (LPS) exposure, is necessary for AVM development in mice [78,100,101,102]. Prenatal or neonatal deletion of *Acvrl1* induces AVMs without exogenous stimulation, due to the naturally highly angiogenic environment of the neonatal mouse brain [103,104]. Using timed deletion of *Eng* in the mouse brain endothelium, deletion occurring at P1–3, P8–10, or P15–17, induced brain AVM formation, although with reduced penetrance at P15–17 (88% and 86% vs. 55%, respectively) [105]. Vascular endothelial growth factor receptor 2 (VEGFR2) expression and activity peaks in the first two weeks after birth in mice, coinciding with the ability to form brain AVMs [105].

Clinical evidence also supports the necessity of an external trigger to initiate brain AVMs. Human brain AVMs, both in sporadic cases and in HHT, are generally considered to be congenital, although there are reports of de novo brain AVMs in both conditions [106,107,108,109]. However, in many of these cases, there exists previous pathology or traumatic incidents, such as traumatic brain injury, seizures, or inflammatory diseases [108,109,110]. These data suggest that de novo brain AVMs, even if seeded by early congenital somatic mutations, require angiogenic stimulation or trauma. It is likely that brain vascular endothelial cells acquire somatic mutations during fetal or early development, when endothelial cells are proliferating due to blood vessel formation. These somatically mutant endothelial cells would lie dormant in the brain until a stimulus such as trauma or growth factors spurs AVM formation.

Mucocutaneous telangiectases also require an external trigger to develop. In *Eng* and *Acvrl1*-induced knockout mice, dermal wounding triggers AVM genesis at the wound site [61,62,66]. The number of telangiectases a person with HHT has is known to increase over time, with clinical evidence of acquired events triggering AVM formation [111,112]. Individuals with HHT report more telangiectases on their dominant hand relative to their nondominant hand, or on their lower lip relative to their upper lip [41]. These areas of the body experience greater physical trauma, briefly stimulating angiogenesis and transforming a quiescent endothelium into a proliferating tissue.

## 16. Blood Flow and AVM Development

Blood flow has been established as a critical factor in combination with biallelic loss of *ENG*, *ACVRL1*, or *SMAD4* function leading to AVM development. *ACVRL1* expression correlates with blood flow in both mice and zebrafish, suggesting a key role of this gene in blood flow in relation to shear stress regulation [113,114]. In *acvrl1*-deficient zebrafish, blood flow is required for AVM formation [113]. Similarly, in vitro vessel-on-a-chip models of HHT require flow to establish an arteriovenous shunt [54].

Animal models have suggested that flow–migration coupling of endothelial cells may play a significant role in AVM development. Endothelial cells in *acvrl1*-deficient zebrafish embryos migrate preferentially away from the heart with the direction of blood flow instead of correctly toward the heart, leading to an accumulation of *acvrl1*-negative endothelial cells in distal vessels [83,113]. AVMs form in *acvrl1* knockout zebrafish embryos from the maintenance of normally transient arteriovenous connections as a response to increased blood flow [113]. In zebrafish, *eng* mutant vein endothelial cells enlarge in response to blood flow in a cell-autonomous manner, leading to enlarged veins to reduce flow resistance and subsequently enlarged arteries to accommodate the higher blood flow [115]. Thus, in zebrafish, the biallelically mutant endothelial cells respond inappropriately to blood flow to cause enlarged distal vessels that maintain high blood flow. Similarly, in mice with brain endothelial cell-specific *Acvrl1* knockout, *Acvrl1*-negative endothelial cells migrate improperly with the direction of flow in mouse retinas [85]. *Eng* knockout endothelial cells also exhibit improper flow–migration coupling, failing to migrate against blood flow and accumulating in the mouse retinal veins [84]. An analysis of HUVECs further suggests that this altered endothelial cell migration involves YAP/TAZ signaling downstream of VEGFR2-integrin stimulated PI3K signaling [85]. Interestingly, loss of venous and capillary *Eng*, but not arterial *Eng*, was established as central to AVM formation in an induced knockout *Eng* mouse retinal AVM model [116]. Thus, the location within the blood vessel of mutant endothelial cells may be important to AVM development and may be dependent on blood flow responses.

Blood flow-induced endothelial cell proliferation may also play a major role in AVM formation. In mice, *Acvrl1* was shown to moderate the suppression of endothelial cell proliferation in response to blood flow and BMP9 stimulation, and *Eng* contributes to this by moderating the suppression of endothelial cell proliferation in response to flow [117]. *acvrl1*-deficient zebrafish embryos show blood flow-dependent endothelial cell proliferation 40–48 h post-fertilization [113]. Moreover, *Smad4* deletion causes retinal AVMs due to dysregulated fluid shear stress response causing improper endothelial cell proliferation, confirming that all three major HHT genes are critical in regulating the endothelial cell response to blood flow [118,119].

HHT genes have critical but distinct roles in the endothelial cell response to flow. Recent work in cell culture models has shown opposing roles for *ACVRL1* and *SMAD4* loss-of-function in decreasing and increasing endothelial sensitivity to shear stress, respectively [120]. This new evidence suggests that AVM development due to *ENG*, *ACVRL1*, or *SMAD4* loss may be caused by different cellular and molecular responses, despite resulting in a similar pathophysiological structure sustaining high blood flow through the arteriovenous shunt. Across multiple models of HHT, including in vitro models and in vivo models of different species, blood flow is required to establish these AVMs, further evidence that a confluence of genetic and non-genetic triggers is required for AVM development.

## 17. Somatic Mutations of Other Genes: The Possibility of Synergistic Mutations

In addition to these physiologic and pathophysiologic triggers, another potential trigger for AVM growth might be the mutation of other genes that synergizes with germline and somatic mutations in a known HHT gene. Although it is very common in cancer to have multiple genes mutated for tumorigenesis [24], there is one prominent example of this in vascular malformations. CCMs acquire both a gain-of-function mutation in *PIK3CA* or *AKT1* in addition to biallelic loss of CCM gene function [28,38,121]. Mutations in *PIK3CA* were shown to act synergistically with CCM loss-of-function to fuel aggressive lesion growth [38]. It is uncertain whether this synergistic mechanism occurs in HHT. To date, no additional gene mutations that synergize with the biallelic loss-of-function have been identified in HHT-associated vascular malformations, despite deep DNA sequencing, albeit on a limited number of samples [56,57,58]. Further investigations using whole exome sequencing with larger sample sizes of brain and lung AVMs will be necessary to fully address this question.

## 18. Implications for Treatment

The contribution of somatic mutations to HHT pathology has important implications for the treatment of HHT. Currently, surgery and embolization remain the primary treatment for life-threatening AVMs. Different drug treatment strategies are under investigation, but they primarily rely on the assumption of haploinsufficiency as the mechanism of vascular malformation development and maintenance. Increasing transcription of the remaining wild-type allele, small molecule stimulation of the BMP9/10 pathway, and synthetic ligand use to increase signaling through the remaining protein [69,122,123,124,125,126] have all been explored.

Although the BMP9/10-ALK1-SMAD1/5/9 signaling axis critical to HHT-associated AVM pathogenesis has been studied quite extensively, the current landscape of drugs used in clinical trials in HHT patients generally consists of broad spectrum tyrosine kinase inhibitors that serve as anti-angiogenic factors [127]. These include pomalidomide, bevacizumab, and pazopanib [127]. The study of these drugs is motivated in part by a desire to expedite treatment in HHT patients by repurposing drugs already approved for other indications. Moreover, most of the current clinical trials focus on controlling bleeding as a primary outcome rather than AVM growth and development [128,129,130,131,132]. Ironically, none of the drugs currently in clinical trials appear to interface directly with the BMP9/10-ALK1-SMAD1/5/9 signaling axis.

By contrast, a new approach sponsored by Diagonal Therapeutics seeks to use ALK1-BMPRII bi-specific antibodies as receptor agonists to increase ALK1-SMAD1/5/9 signaling [133]. This approach directly targets the critical signaling axis that is disrupted in the endothelial cells of HHT-associated AVMs by seeking to augment signaling through the remaining ALK1 receptor protein [133]. However, the genotype of the patient may be a critical factor in the effectiveness of the drug. Increasing signaling of ALK1 could be effective; however, it would require ALK1 protein expression in the endothelial cells of the AVM. A recent study found that the overexpression of ALK1 in an *Acvrl1-* or *Eng*-inducible knockout mouse model inhibited AVM development in skin-wound and developing retinal models [134]. Conversely, the overexpression of ENG could not prevent AVMs in *Acvrl1-* or *Eng*-deficient mice [134]. Thus, in *ENG* patients, the bi-specific antibody strategy may work as intended, since ALK1 expression is not affected. *ACVRL1* patients may present a greater challenge. The bulk of the endothelial cells within the AVM would be heterozygous, indicating that this approach of augmenting signaling through the remaining receptor protein may be efficacious. However, a minority of cells in the AVM will be somatically mutant and thus lack any functional ALK1 protein. These homozygous mutant cells would be unresponsive to the drug.

The efficacy of these new strategies will depend on the contribution of somatically mutant versus non-somatically mutant cells to the maintenance of established AVMs. If the underlying heterozygous endothelium exerts the most influence, then these strategies may be effective. However, if biallelically mutant endothelial cells play a major role in maintaining AVMs, these strategies may be ineffective, since the relevant endothelial cells will express little to none of the HHT gene. There is evidence that biallelically mutant endothelial cells exert cell non-autonomous effects on the surrounding endothelium, contributing to the development and growth of AVMs. In a mouse retinal AVM model, mosaic endothelial cell deletion of *Eng* caused increased proliferation of both *Eng* loss-of-function cells and their wild-type neighbors within AVMs [84]. Similarly, in a brain AVM model, mosaic loss of *Acvrl1* caused increased proliferation of both *ACVRL1* loss-of-function and wild-type cells within the AVMs [79]. This evidence may indicate that the biallelically mutant endothelial cells within the lesional vessel must also be targeted to fully treat an AVM. Recent evidence from zebrafish that blood flow stimulates *acvrl1* transcription in a ligand-dependent manner has led to the suggestion that this property may be exploited to increase *ACVRL1* expression and function in HHT patients [125]. Further investigation into this strategy must consider the already increased flow found in AVMs and the effectiveness of this strategy in *ENG* versus *ACVRL1* patients. Thus, both the germline and somatic genotype of the patient’s vascular malformations, and their relative contributions to the pathology of the lesion, will be primary considerations for future treatment considerations.

## 19. Conclusions

Over the past decades, somatic mutation has emerged as a critical determinant of vascular malformation pathogenesis in multiple forms of sporadic lesions and inherited disorders. The recent evidence from human HHT-associated vascular malformation tissues supports a role for second-hit somatic mutation in the pathogenesis of HHT-associated AVMs. These data, together with data from preclinical models, support a multifactorial model of HHT-associated vascular malformation development involving somatic mutation, cell autonomous, and cell non-autonomous processes in the generation of the AVM. While the exact processes of HHT-associated AVM pathogenesis have not yet been elucidated, the role of somatic mutation in this process will be of critical importance to future investigations into drug treatments for HHT.

## Abbreviations

The following abbreviations are used in this manuscript:

AVM	Arteriovenous malformation
BMP	Bone morphogenetic protein
CCM	Cerebral cavernous malformation
HHT	Hereditary hemorrhagic telangiectasia
HUVEC	Human umbilical vein endothelial cells
IGV	Integrative Genomics Viewer
JP-HHT	Juvenile polyposis–HHT
LPS	Lipopolysaccharide
PCR	Polymerase chain reaction
PAH	Pulmonary arterial hypertension
TGFB	Transforming growth factor beta
UV	Ultraviolet
